# Hemodynamic responses and functional aerobic capacity in adults: insights from NASA method estimated VO₂

**DOI:** 10.3389/fresc.2026.1731940

**Published:** 2026-02-23

**Authors:** Leonardo Arzayus-Patiño, Jenifer Rodriguez-Castro, Jhonatan Betancourt-Peña, Juan C. Avila-Valencia, Vicente Benavides-Cordoba

**Affiliations:** 1 Faculty of Health, Physiotherapy Program, Universidad Santiago de Cali, Cali, Colombia; 2 Facultad de Ciencias del Movimiento, Fundación Universitaria de Ciencias de la Salud FUCS, Bogotá, Colombia; 3 Facultad de Salud y Rehabilitación, Institución Universitaria Escuela Nacional del Deporte. Escuela de Rehabilitación Humana Universidad del Valle Cali, Cali, Colombia; 4 Facultad de Salud y Rehabilitación, Institución Universitaria Escuela Nacional del Deporte. Clínica de Occidente S.A. Cali, Cali, Colombia,; 5 Facultad de Salud, Departamento de Ciencias Fisiológicas, Universidad del Valle. Cali, Cali, Colombia

**Keywords:** blood pressure, cardiorespiratory fitness, exercise test, heartrate, hemodynamic gain index, oxygen consumption, sit-to-stand test, two-minute step test

## Abstract

**Introduction:**

Cardiorespiratory fitness, expressed as VO₂max, is a key indicator of cardiovascular and pulmonary health. Field-based tests such as the Sit-to-Stand (STS) and Two-Minute Step Test (2MST) provide accessible alternatives for evaluating functional capacity in rehabilitation settings. This study evaluated hemodynamic responses and estimated aerobic capacity from these functional tests and explored their associations using the NASA non-exercise VO₂max equation.

**Materials and methods:**

A cross-sectional correlational study (2023–2024) was conducted in healthy adults from Cali and Bogotá, Colombia, under STROBE guidelines. Participants completed the International Physical Activity Questionnaire (IPAQ), three Sit-to-Stand variants (5-repetition, 30-second, and 1-minute), and the 2MST. Heart rate, blood pressure, oxygen saturation, and perceived exertion were recorded before and after each test, along with recovery at 1 and 2 minutes. The Hemodynamic Gain Index (HGI) was calculated, and VO₂max was estimated using the NASA formula.

**Results:**

A total of 260 adults participated. Hypertension (30.8%) and diabetes (23.8%) were the most frequent comorbidities. All tests increased heart rate and blood pressure significantly (*p* < 0.001), with the greatest responses in the 2MST and 1-min STS, while oxygen saturation remained stable. The 2MST and 1-min STS produced the highest HGI and exertion levels. VO₂max correlated negatively with age and BMI and positively with height and functional performance, especially the 30-s STS. HGI showed modest correlations with VO₂ estimates in STS tests (r = 0.21–0.28).

**Conclusion:**

The 2MST elicited stronger hemodynamic responses, while STS variants were more closely associated with estimated VO₂max, supporting their combined use for efficient assessment of functional capacity, which is relevant for monitoring and guiding rehabilitation programs.

## 1 Introduction

The evaluation of functional capacity and the hemodynamic response to exercise represents a cornerstone in both research and clinical practice, given its impact on cardiovascular health and its predictive value for short- and long-term outcomes. Low levels of cardiorespiratory fitness, measured through maximal oxygen consumption (VO₂max), have been consistently associated with an increased risk of cardiovascular disease and mortality (1). Recent studies confirm that even moderate reductions in VO₂max are linked to increased incidence of hypertension, metabolic syndrome, and all-cause mortality, reinforcing its role as a critical biomarker of health status (2, 3).

Conventional exercise tests, such as cardiopulmonary exercise testing, provide precise measurements by integrating the responses of the cardiovascular, respiratory, and muscular systems, and are considered the gold standard for assessing aerobic capacity (4). However, their implementation is limited by the requirement for specialized equipment, trained personnel, and high costs, which restricts their applicability in community or population-based settings (5).

In this context, field-based functional tests such as the Sit-to-Stand Test (STS) in its different variants and the Two-Minute Step Test (2MST) have gained relevance due to their ease of application, safety, and low cost. The STS has been widely used as a field test to assess functional capacity (6), in healthy individuals (7) and in individuals with respiratory and chronic diseases (8, 9), as it requires minimal equipment and is associated with more complex measures of functional capacity. Similarly, the 2MST has been described as a low-cost, reproducible tool suitable for estimating aerobic capacity in resource-limited settings (10). Beyond their utility as physical performance tests, both elicit cardiovascular responses that can be analyzed through integrative indicators, among which the Hemodynamic Gain Index (HGI) stands out. This index combines heart rate and blood pressure, providing a global measure of the relative hemodynamic effort during exercise and offering a complementary perspective to the evaluation of functional capacity (11).

In this same context, the indirect estimation of VO₂max through non-invasive predictive models, such as the one developed by Jurca and colleagues in the NASA cohort, complements the information provided by the HGI, as it integrates simple variables sex, age, body mass index, resting heart rate, and self-reported physical activity level, to practically estimate cardiorespiratory fitness. This model has demonstrated multiple correlations ranging from 0.81 to 0.76 and standard errors of estimation between 1.45 and 1.97 METs, establishing itself as a valuable tool in both research and public health settings (12).This approach provides a practical alternative for estimating oxygen consumption and functional aerobic capacity, supporting its application in research and public health contexts.

Nevertheless, knowledge gaps remain regarding how short-duration functional tests relate to immediate hemodynamic responses and to aerobic capacity estimated through non-invasive methods. While both the HGI and predictive formulas derived from the NASA cohort offer useful and accessible approaches, it is still unclear to what extent HGI values obtained from tests such as the Sit-to-Stand Test or the Two-Minute Step Test consistently reflect the aerobic capacity estimated by these models. Deepening our understanding of these associations is crucial to more precisely elucidate the physiological mechanisms underlying functional performance and to establish the value of these simple tools in predicting cardiorespiratory fitness. Accordingly, the focus of this approach lies on exploring the relationship between hemodynamic responses elicited by simple functional tests and aerobic capacity estimated through non-invasive models, particularly in contexts where cardiopulmonary exercise testing is not routinely available.

In this context, the present study aimed to evaluate the hemodynamic responses and functional aerobic capacity derived from different functional tests, and to explore their associations using VO₂ estimates obtained through the NASA formula.

## 2 Materials and methods

### 2.1 Study design and participants

This cross-sectional and correlational study was conducted between 2023 and 2024 in Cali and Bogotá (Colombia), following the STROBE guidelines (28). Participants were recruited using a convenience sampling approach, and the final sample comprised all individuals from the general population who expressed willingness to participate and fulfilled the inclusion criteria during the data collection period. A total of 260 participants were included in the final analysis.The study was approved by the Ethics Committee of the Fundación Universitaria de Ciencias de la Salud (FCM-I-032-2024), and all participants signed an informed consent form.

Inclusion criteria were: age ≥18 years, the ability to perform sit-to-stand and stepping movements, and self-reported good health. “Good health” was defined as the absence of functional limitations that could affect performance in the functional tests. Participants with chronic conditions were eligible only if these conditions were clinically stable or well controlled at the time of assessment. Exclusion criteria included a body mass index (BMI) > 35 kg/m^2^ and a history of recent respiratory, musculoskeletal, or neuromuscular conditions that could interfere with performance in the functional tests.

### 2.2 Measurements

Participants attended a single evaluation session in which anthropometric variables and physical activity levels were recorded. Physical activity was assessed with the short version of the International Physical Activity Questionnaire (IPAQ). Functional performance was evaluated with the Sit-to-Stand Test in three variants: 1-minute (STS-1 min), 30-second (STS-30s), and 5-repetition (STS-5rep), and with the 2mST.

Vital signs, including heart rate (HR), systolic blood pressure (SBP), diastolic blood pressure (DBP), oxygen saturation (SpO₂), respiratory rate and perceived exertion were measured before, immediately after each functional test, and one minute after completing the tests. Oxygen saturation was obtained with a portable pulse oximeter (Nonin, USA), while SBP and DBP were measured using a digital sphygmomanometer (Welch Allyn, USA). Respiratory rate was assessed manually by observing the rise and fall of the chest over a full 60 s while the participant was seated and at rest, ensuring minimal movement and without alerting them to the measurement to avoid altering their breathing pattern. Perceived exertion was assessed using the modified Borg scale (0–10) separately for dyspnea and fatigue. All functional tests were performed in a fixed order, starting with the STS-1 min, followed by the STS-30s and STS-5rep, and concluding with the 2-minute step test. A standardized 20-minute rest period was provided between tests to ensure adequate cardiovascular recovery and to minimize potential carryover effects.

### 2.3 Functional tests

All functional tests were conducted by the same group of trained evaluators. Prior to data collection, the evaluators completed training sessions in which they recorded three demonstration videos of the field tests using a pilot participant. The videos were reviewed to confirm adherence to the study protocol, and data collection commenced only after validation was approved.

*STS:* Participants performed the STS test using a standard chair (43–46 cm seat height, with back support and no armrests), which was placed against a wall to ensure stability and prevent movement during execution. They were instructed to sit upright with hips and knees flexed at approximately 90°, feet placed flat on the floor and shoulder-width apart, and arms crossed on the chest to avoid the use of the upper limbs for assistance. Clear standardized instructions were provided, and a demonstration was given before each attempt to ensure proper technique. For the STS-1 min and STS-30s, the outcome measure was the total number of repetitions completed within the specified time, with each repetition defined as standing up fully extended and then returning to the initial seated position. For the STS-5rep, the variable of interest was the time in seconds required to complete five full repetitions consecutively, recorded with a digital stopwatch. Test administrators provided verbal encouragement to maximize participant effort, and rest was allowed between trials if needed. Heart rate, blood pressure, and perceived exertion were monitored before and immediately after the test to capture the acute hemodynamic response.

*2mST:* The 2MST was conducted as a submaximal field test designed to evaluate functional aerobic capacity and cardiovascular endurance. Participants were instructed to march in place for two minutes, lifting the knees to a target height corresponding to the midpoint between the superior border of the patella and the iliac crest. A visual marker was placed on a wall or adjustable stand to help participants maintain the correct knee elevation throughout the test. The total number of steps performed with the right knee was recorded as the final score, following standardized protocols. The test was administered indoors on a flat surface, with sufficient space to allow safe movement, and participants were reminded to maintain a steady rhythm according to their tolerance. Continuous monitoring ensured safety, and the test was stopped if any adverse signs or symptoms (e.g., dizziness, chest pain, or excessive dyspnea) occurred. Vital signs, including heart rate, blood pressure, and oxygen saturation, were measured immediately before and after the test, and the perceived exertion was assessed using the Borg scale.

### 2.4 Hemodynamic index of effort (HGI)

The HGI was calculated to assess cardiovascular responses to exertion and to provide an integrative measure of hemodynamic stress during exercise. It was defined as: HGI=([HR_peak×SBP_peak] – [HR_rest×SBP_rest])/(HR_rest×SBP_rest) where HR denotes heart rate and SBP systolic blood pressure, both measured at rest and immediately after exercise. Resting values were obtained after at least five minutes of seated rest, while peak values were recorded within the first minute following exercise termination to capture the acute cardiovascular response. This index reflects the relative increase in the rate–pressure product, a recognized surrogate of myocardial oxygen demand, thus providing a normalized indicator of cardiovascular effort during functional testing (11).

### 2.5 Estimation of aerobic capacity (NASA method)

Aerobic capacity was estimated using the NASA non-exercise regression method, which predicts VO₂max and metabolic equivalents (METs). The model includes sex, age, BMI, resting heart rate, and self-reported physical activity level (PAL). The equation for METs was: MET = [sex×(2.77)-age×(0.10)-BMI×(0.17)-HRr ×(0.03)+PAL×(1.0)] + 18.07. VO₂max was derived by multiplying MET by 3.5 mL·kg^−1^·min^−1^ (12).

### 2.6 Statistical analysis

All analyses were performed using GraphPad Prism 8.0 (GraphPad Software, San Diego, CA, USA). Descriptive statistics were expressed as means and standard deviations (SD) for continuous variables and as absolute and relative frequencies for categorical variables. Normality of the data was verified using the Kolmogorov–Smirnov test, and since the variables followed a parametric distribution, parametric tests were applied throughout.

Comparisons of baseline, final, and recovery values of vital signs across the functional tests were performed using paired t-tests. To compare the HGI among the four functional tests (STS-1 min, STS-30s, STS-5rep, and 2MST), one-way analysis of variance (ANOVA) was applied, followed by Bonferroni's *post hoc* test for pairwise comparisons. Correlations between functional test performance, estimated aerobic capacity (NASA VO₂ and METs), and the HGI were assessed using Pearson's correlation coefficient. Effect sizes were reported as mean differences with 95% confidence intervals (CI) for comparisons, and as correlation coefficients (r) for associations. A significance level of *p* < 0.05 was considered statistically significant.

## 3 Results

Table 1 presents the sociodemographic, clinical, and anthropometric characteristics of the study population. The sample consisted of 260 participants, with a relatively balanced sex distribution (53.1% women and 46.9% men). Regarding marital status, 43.5% reported being married, 46.9% living in a common-law relationship, 8.8% single, and 0.8% widowed.

**Table 1:** General characteristics of the study population.

Variable	Category	n (%) or Mean (SD)
Sex	Female	138 (53.1)
	Male	122 (46.9)
Marital status	Single	23 (8.8)
	Married	113 (43.5)
	Common-law	122 (46.9)
	Widowed	2 (0.8)
Currently employed	Yes	111 (42.7)
	No	149 (57.3)
Family history	Yes	188 (72.3)
	No	72 (27.7)
Hypertension	Yes	80 (30.8)
	No	180 (69.2)
Diabetes mellitus	Yes	62 (23.8)
	No	198 (76.2)
Coronary artery disease	Yes	19 (7.3)
	No	241 (92.7)
Alcohol consumption	Yes	33 (12.7)
	No	227 (87.3)
Smoking	Yes	32 (12.3)
	No	228 (87.7)
Physical activity level	High	70 (26.9)
	Moderate	135 (51.9)
	Low	55 (21.2)
Age (years)		42.08 (18.55)
Weight (kg)		68.15 (11.83)
Height (m)		1.63 (0.09)
BMI (kg/m^2^)		25.53 (3.82)

Concerning employment status, 42.7% reported being employed at the time of the study, while 57.3% were not. A family history of disease was observed in 72.3% of individuals. Regarding comorbidities, arterial hypertension was reported in 30.8%, diabetes mellitus in 23.8%, and coronary artery disease in 7.3%.

Regarding habits, 12.7% reported alcohol consumption and 12.3% reported smoking. Physical activity level was classified as high in 26.9% of participants, moderate in 51.9%, and low in 21.2%. Anthropometric variables showed a mean age of 42.08 years (SD = 18.55), an average weight of 68.15 kg (SD = 11.83), a height of 1.63 m (SD = 0.09), and a mean body mass index (BMI) of 25.53 kg/m^2^ (SD = 3.82).

Table 2 summarizes the vital signs and perceived exertion during the functional tests. Heart rate increased noticeably at the end of each test, with the highest values observed in the Two-Minute Step Test (110.82 ± 19.51 bpm) and the One-Minute Sit-to-Stand Test (108.02 ± 19.32 bpm). During recovery, heart rate declined but remained above baseline levels.

**Table 2:** Vital signs and perceived exertion in different functional tests.

Signs/Symptoms	Sit-to-Stand 1 min	Sit-to-Stand 30 s	Sit-to-Stand 5 reps	2-min Step Test
Heart rate (bpm)				
Baseline	77.27 ± 9.77	77.52 ± 11.22	78.00 ± 11.63	79.20 ± 11.85
Final	108.02 ± 19.32	101.82 ± 17.42	91.11 ± 13.89	110.82 ± 19.51
Recovery	89.34 ± 15.02	86.11 ± 13.32	81.84 ± 12.56	93.57 ± 17.11
Respiratory rate (breaths/min)				
Baseline	17.07 ± 3.54	17.51 ± 3.44	17.41 ± 3.53	17.30 ± 3.35
Final	22.59 ± 4.82	21.85 ± 5.16	20.30 ± 4.61	23.62 ± 5.57
Recovery	19.27 ± 4.02	18.89 ± 4.26	18.24 ± 3.94	19.69 ± 4.17
Systolic blood pressure (mmHg)				
Baseline	118.35 ± 9.00	118.62 ± 9.04	118.82 ± 8.35	118.39 ± 8.56
Final	128.57 ± 11.52	127.48 ± 11.75	123.93 ± 10.86	130.24 ± 12.33
Recovery	122.15 ± 10.96	121.27 ± 11.21	119.56 ± 9.84	123.39 ± 11.00
Diastolic blood pressure (mmHg)				
Baseline	77.87 ± 8.56	77.54 ± 8.48	78.06 ± 8.78	78.19 ± 8.38
Final	82.41 ± 9.30	81.76 ± 9.65	80.38 ± 8.67	84.65 ± 9.39
Recovery	78.98 ± 8.98	78.80 ± 8.67	78.16 ± 8.74	79.69 ± 9.27
Oxygen saturation (%)				
Baseline	94.86 ± 2.39	94.96 ± 2.34	94.99 ± 2.28	94.95 ± 2.28
Final	94.84 ± 3.16	94.83 ± 2.21	94.84 ± 2.25	94.96 ± 2.10
Recovery	95.11 ± 2.26	94.92 ± 2.22	94.74 ± 2.33	94.95 ± 2.13
HGI				
Mean ± SD	0.54 ± 0.33	0.44 ± 0.32	0.23 ± 0.20	0.56 ± 0.35
Range (min–max)	−0.14–1.84	−0.18–2.02	−0.14–0.99	−0.14–2.47
Borg dyspnea (0–10)				
Baseline	0.22 ± 0.76	0.35 ± 0.90	0.40 ± 1.08	0.33 ± 0.88
Final	2.29 ± 1.83	1.70 ± 1.58	0.98 ± 1.30	3.07 ± 2.06
Recovery	1.00 ± 1.25	0.88 ± 1.31	0.60 ± 1.01	1.43 ± 1.41
Borg fatigue (0–10)				
Baseline	0.42 ± 1.06	0.49 ± 1.09	0.52 ± 1.13	0.45 ± 1.04
Final	2.58 ± 2.08	1.96 ± 1.75	1.26 ± 1.48	3.96 ± 2.33
Recovery	1.21 ± 1.40	1.10 ± 1.49	0.75 ± 1.20	2.07 ± 1.77

Respiratory rate followed a similar pattern, rising at the end of the tests and peaking in the Two-Minute Step Test (23.62 ± 5.57 breaths/min). In the recovery phase, values returned close to those recorded at rest.

Systolic blood pressure reached its highest value in the Two-Minute Step Test (130.24 ± 12.33 mmHg), while diastolic pressure showed smaller increases across all tests, also reaching its maximum in the Two-Minute Step Test (84.65 ± 9.39 mmHg). Oxygen saturation remained stable throughout, with no clinically relevant reductions, and values consistently ranged between 94% and 95%.

The HGI showed higher responses in the Two-Minute Step Test (0.56 ± 0.35) and the One-Minute Sit-to-Stand Test (0.54 ± 0.33), compared to the 30-second Sit-to-Stand Test (0.44 ± 0.32) and the Five-Repetition Sit-to-Stand Test (0.23 ± 0.20).

Perceived exertion increased during the effort phase. On the Borg dyspnea scale, the highest scores were found in the Two-Minute Step Test (3.07 ± 2.06) and the One-Minute Sit-to-Stand Test (2.29 ± 1.83). On the Borg fatigue scale, scores were also highest in the Two-Minute Step Test (3.96 ± 2.33) and the One-Minute Sit-to-Stand Test (2.58 ± 2.08). In all cases, both dyspnea and fatigue scores decreased during recovery but did not return to initial baseline levels.

**Figure 1:**
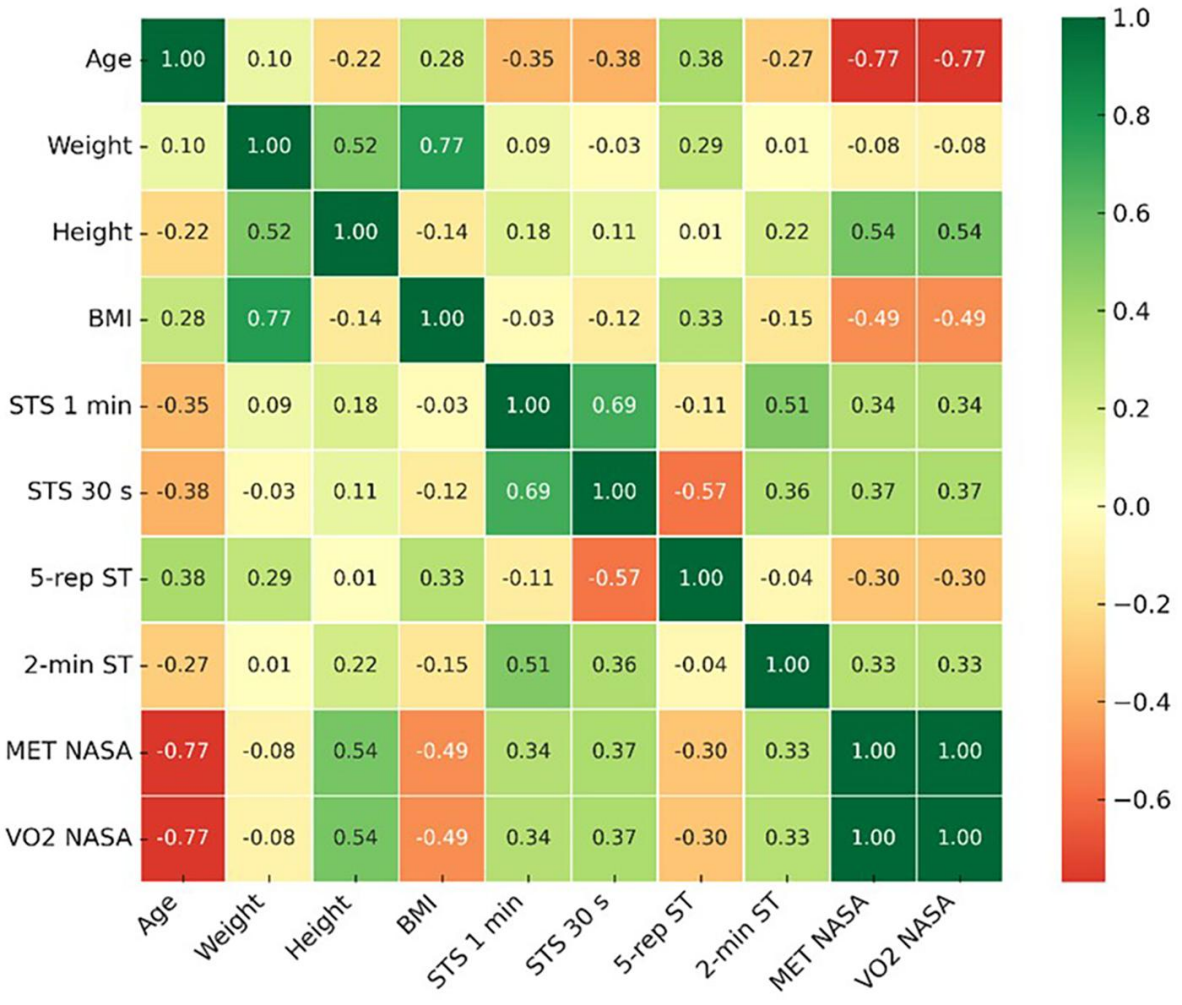
Correlation between functional tests and aerobic capacity estimated by NASA MET and VO₂.

Figure 1 shows the correlation map between sociodemographic and anthropometric variables and the functional tests with aerobic capacity estimates based on NASA METs and NASA VO₂. Strong and significant negative correlations were observed between age and both reference variables (NASA MET: r = −0.771; NASA VO₂: r = −0.771; *p* < 0.01). Similarly, BMI showed a moderate negative correlation with NASA MET (r = −0.487; *p* < 0.01) and NASA VO₂ (r = −0.487; *p* < 0.01). In contrast, height displayed a moderate positive correlation with these variables (r = 0.538; *p* < 0.01).

Likewise, the functional tests showed positive and significant associations with estimated aerobic capacity. The highest correlation was observed with the 30-second STS (r = 0.367; *p* < 0.01), followed by the 1-minute STS (r = 0.335; *p* < 0.01) and the 2-minute step test (r = 0.330; *p* < 0.01). In contrast, the 5-repetition STS time showed a moderate negative correlation (r = −0.302; *p* < 0.01), indicating that longer completion times are associated with lower NASA MET and VO₂ values.

When comparing the HGI across the four functional tests, significant differences were observed. The 1-min STS demonstrated a higher HGI than both the 30-s STS (*Δ* = 0.10; 95% CI: 0.04 to 0.17; *p* < 0.001) and the 5-repetition STS (*Δ* = 0.30; 95% CI: 0.24 to 0.36; *p* < 0.0001). No significant difference was found between the 1-min STS and the 2-min step test (*Δ* = –0.03; 95% CI: −0.10 to 0.03; *p* = 0.596).

**Figure 2:**
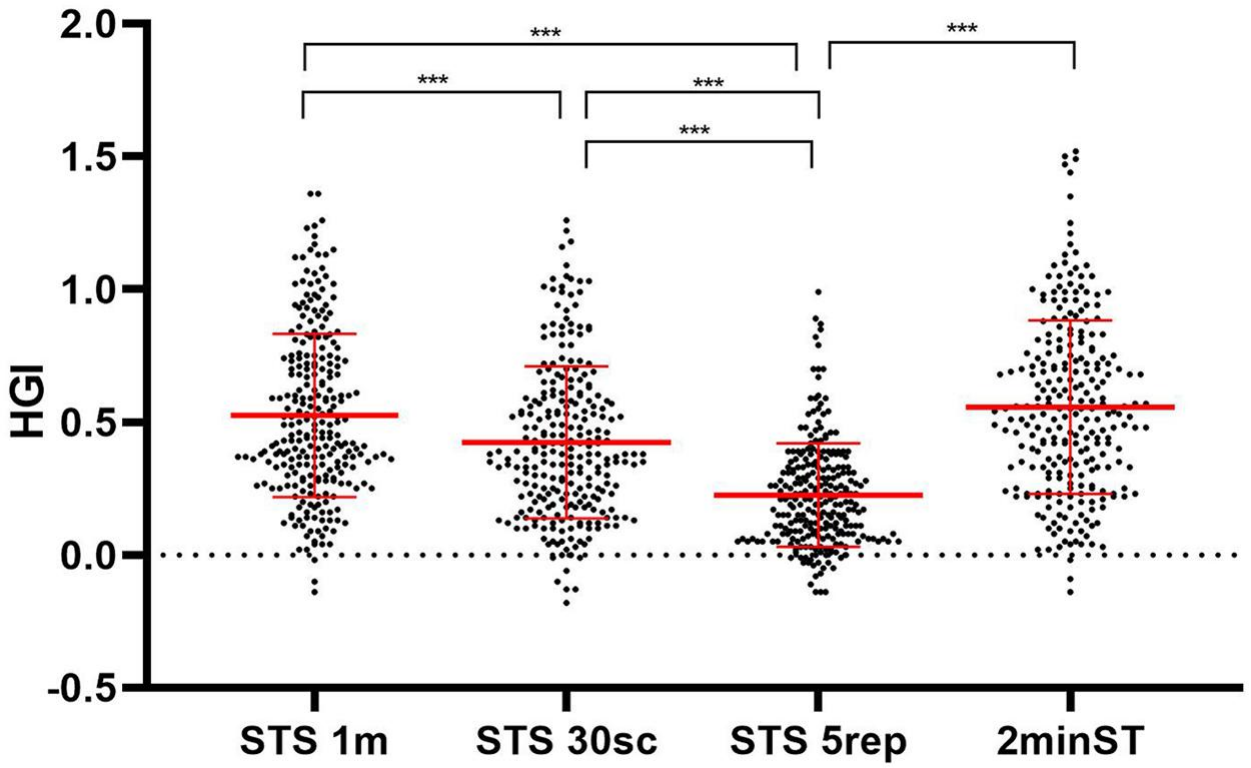
Comparison of the hemodynamic effort index across the four functional tests.

The 30-s STS showed a significantly higher HGI compared with the 5-repetition STS (*Δ* = 0.20; 95% CI: 0.13 to 0.26; *p* < 0.0001), but a lower HGI than the 2-min step test (*Δ* = –0.13; 95% CI: −0.20 to −0.07; *p* < 0.0001). The 5-repetition STS yielded the lowest HGI, being significantly lower than all other tests (*Δ* = –0.33; 95% CI: −0.40 to −0.27; *p* < 0.0001) (Figure 2).

**Figure 3:**
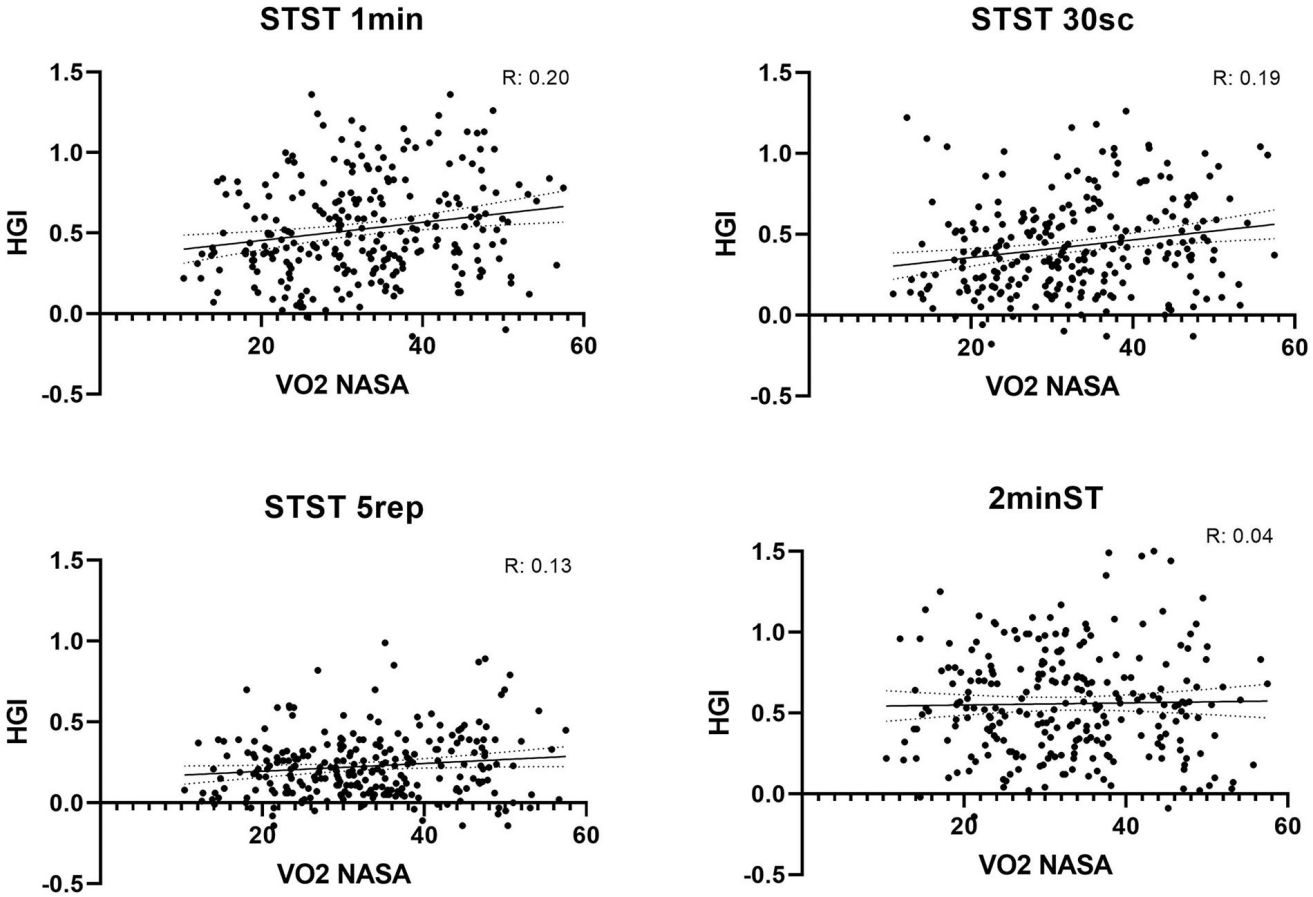
Correlation between estimated VO₂ using the NASA formula and the hemodynamic effort index derived from different functional tests.

When analyzing the relationship between the VO₂ estimated using the NASA formula and the HGI calculated from the hemodynamic response across the different functional tests, low-magnitude but statistically significant positive correlations were identified in three of them. The 1-min STS showed the strongest correlation (r = 0.201; *p* = 0.001), followed by the 30-s STS (r = 0.197; *p* = 0.001), whereas the 5-repetition STS demonstrated a weaker yet significant association (r = 0.137; *p* = 0.028). In contrast, the HGI derived from the 2-min step test did not show a significant relationship with VO₂ estimated using the NASA formula (r = 0.047; *p* = 0.455). Overall, these findings suggest that HGI values obtained from the STS variants exhibit a more consistent relationship with estimated VO₂, whereas the 2MST did not show an association with this physiological variable (Figure 3).

## 4 Discussion

The results of this study provide evidence on the relationship between different short-duration functional tests, immediate hemodynamic responses, and the estimation of aerobic capacity using a non-invasive model based on the NASA formula. Collectively, these findings contribute to a deeper understanding of exercise physiology in contexts that require simple, reproducible, and low-cost tools to assess cardiorespiratory fitness. Recent systematic reviews emphasize that functional field tests are increasingly being used as valid alternatives in both rehabilitation and preventive programs, particularly when laboratory assessments are not available (13).

From a physiological perspective, the observed increases in heart rate, respiratory rate, and blood pressure during testing are consistent with sympathetic nervous system activation and the integrative cardiovascular response to dynamic exercise (14). The fact that the 2MST and the 1-min STS elicited the most pronounced increases in heart rate and blood pressure reflects their greater cardiovascular demand, arising from both the duration of the load and the volume of muscle engaged in the repetitive movement (15). This observation aligns with previous findings in clinical populations, such as patients with arterial hypertension, where the 2MST has been shown to elicit a significant cardiovascular response (16).

In contrast, shorter-duration tests, such as the STS-30s and STS-5rep, tend to elicit a more attenuated hemodynamic response, likely because their brief duration limits the ability of the body to reach a stable physiological and hemodynamic state. Under these conditions, sympathetic activation and increases in cardiac output begin, but do not fully consolidate before the effort concludes, explaining the relatively smaller increases observed in cardiovascular variables (17).

The stability of oxygen saturation across all protocols indicates that, in this healthy general population, the respiratory system and pulmonary diffusion are capable of maintaining adequate gas exchange even under exercise conditions, which aligns with findings from other functional tests, such as the six-minute walk test, where healthy individuals also do not experience desaturation (18). In patients with COPD, it has been shown that the one-minute sit-to-stand test (1STST) is a useful tool for detecting exercise-induced desaturation. Given its short duration and minimal resource requirements, these tests, can be applied in outpatient settings to routinely assess exercise capacity and desaturation during exertion in this population, reinforcing its value as a practical tool for functional evaluation and clinical follow-up (16).

The analysis of the HGI represents a novel contribution by integrating heart rate and blood pressure into a single metric of relative hemodynamic load. Our results showed that the 2MST and the 1-min STS produced higher HGI values compared to the 30-s STS and the 5-repetition STS. Physiologically, this suggests that these test variants impose greater cardiovascular stress, which may result not only from the total duration of exercise exposure but also from continuous muscle recruitment and the need to maintain a constant contraction rhythm, leading to increased chronotropic and inotropic activation. Studies have shown that under greater load or muscular fatigue, the body adapts its motor strategy to redistribute effort across muscle groups, involving changes in muscle activation and, consequently, cardiovascular demand (19).

Functionally, these differences are meaningful. The HGI could be interpreted as an indirect indicator of cardiovascular efficiency: a higher index, within a physiological range, reflects the capacity of the hemodynamic system to adapt to relatively simple physical loads. HGI provides an integrated assessment of the cardiovascular response to exercise in a single metric, offering a non-invasive and sensitive measure of cardiovascular function that has been independently associated with mortality. The product of heart rate and systolic blood pressure, known as the rate–pressure product, serves as an indirect measure of myocardial oxygen consumption and overall cardiac function (20, 21). Consistent with this concept, recent evidence in patients with heart failure with preserved ejection fraction has shown that higher HGI values are associated with greater exercise capacity, more favorable biomarker profiles, and improved prognosis, supporting its relevance as an integrative marker of cardiovascular reserve during exercise (11). Therefore, the HGI reflects the net capacity of the cardiovascular system to generate adequate blood flow in response to physical demands, providing added value in interpreting the hemodynamic response during functional tests such as the STS variants and the 2MST.

Regarding the VO₂max estimated using the NASA equation, the STS variants appear to offer more consistent information for its estimation, whereas the 2MST, despite producing greater hemodynamic stress, did not show a significant correlation with the estimated VO₂. This finding aligns with the observation that the relationship between cardiovascular load and oxygen consumption is not always linear, as biomechanical and neuromuscular adjustments observed during fatigue, such as changes in contact and propulsion times without altering total mechanical work, suggest that the body modifies movement strategies to maintain mechanical output, thereby modulating the physiological response to exertion (22). However, the 2MST has been primarily recommended for adults aged ≥65 years, particularly as a simple and safe functional measure in older populations and in settings where space or walking-based tests are not feasible, whereas STS-based tests may be less suitable in individuals with balance impairment or increased risk of falls (23).

Negative correlations between age and estimated aerobic capacity are consistent with the progressive physiological decline in cardiovascular and musculoskeletal function that occurs with aging (24). Similarly, the impact of body mass index reflects how excess weight limits the mechanical efficiency of movement and increases the relative demand of effort, thereby reducing performance in functional tests (25), as in VO₂max measurement (26). In contrast, the positive association between height and estimated VO₂ values may be explained by the greater body surface area and relative muscle mass in taller individuals, which contributes to a higher oxygen consumption potential.

In clinical and community practice, these findings have several implications. First, they confirm that simple tests such as the STS and 2MST not only allow functional performance assessment but also elicit measurable physiological responses that provide insight into cardiovascular reserve. Moreover, these tests may be useful in other contexts such as pulmonary rehabilitation, where monitoring functional progress and hemodynamic adaptation is essential for evaluating intervention effectiveness (27).Second, incorporating the HGI alongside traditional variables could enhance the sensitivity of these tests to identify individuals with reduced hemodynamic adaptation capacity, even in the absence of overt disease. Finally, the association between STS performance and estimated VO₂ reinforces the value of these tests as cost-effective approaches for assessing cardiorespiratory fitness in public health, rehabilitation, and healthy lifestyle promotion contexts.

Key limitations of this study include the small sample size, the indirect estimation of VO₂max using the NASA equation, and the exclusive inclusion of a healthy population, which limits the generalizability of the findings to clinical populations. Likewise, conducting the tests in a single controlled setting may not fully reflect their application conditions in other contexts. These limitations were mitigated through the use of a standardized functional testing design, validated protocols, and the comparison of multiple STS and 2MST variants to ensure internal consistency. Additionally, the NASA equation was chosen for its validated robustness in the general population, and the experimental context was thoroughly documented to facilitate future replication in different populations and settings.

Overall, the findings of this study contribute toward a more integrative framework in which exercise physiology, cardiovascular responses, and functional estimation of aerobic capacity are jointly analyzed. This approach supports the selection of simple and reproducible tools adaptable to diverse clinical and community settings, with the potential to optimize early identification of cardiorespiratory limitations and guide preventive or rehabilitative interventions.

## 5 Conclusion

The findings suggest that hemodynamic responses, assessed through the Hemodynamic Gain Index (HGI), tend to be greater in longer-duration tests such as the STS-1 min and the 2MST, reflecting a higher cardiovascular demand. Conversely, Sit-to-Stand variants showed more consistent associations with VO₂max estimation using the NASA equation, indicating their potential as indirect approximations of aerobic capacity. This suggests that integrating HGI with non-invasive VO₂ estimates could provide a valuable complementary approach for understanding and monitoring exercise responses, thereby supporting more comprehensive and accessible functional assessments.

## Data Availability

The raw data supporting the conclusions of this article will be made available by the authors, without undue reservation.
